# Moral dilemma(s) in human papillomavirus vaccination – revisiting the role of the herd effect

**DOI:** 10.2807/1560-7917.ES.2021.26.50.2101154

**Published:** 2021-12-16

**Authors:** Matti Lehtinen, Ville N. Pimenoff

**Affiliations:** 1 FICAN-Mid, Tampere University Hospital, Tampere, Finland; 2 Department of Laboratory Medicine, Karolinska Institute, Stockholm, Sweden

**Keywords:** HPV vaccination, vaccination coverage, gender-neutral, shortage, herd effect

In this *Eurosurveillance* issue, two articles by Colzani et al. [1] and Ortu et al. [2], raise the question of who should be prioritised in vaccination policy when there is a temporary global shortage of vaccines. These articles on human papillomavirus (HPV) are a reminder that discussions pertaining to vaccination policies are not confined to coronavirus disease (COVID-19). Nonetheless, lessons learnt from the establishment of HPV vaccination are generalisable and valuable for the implementation and monitoring of most vaccination programmes, including those against COVID-19, worldwide.

Colzani et al. [1] use the eradication of rubella as an example of beneficial gender-neutral vaccination. Indeed, together with cost-efficiency of offering HPV vaccination to both girls and boys, already at moderate coverage, the subsequently achieved herd effect (i.e. indirect protection to the unvaccinated in a population) is very important. In the past, eradication attempts with girls-only rubella virus vaccination failed in the Nordic countries. However, rubella – as well as mumps – was eradicated from the Nordic countries in just 15 years (between 1982 and 1997) after the launch of a gender-neutral measles-mumps-rubella (MMR) vaccination programme [3]. Girls-only rubella vaccination with 70% coverage in the 1970s only moved the peak-age of rubella incidence to fertile-aged women without any effect on the incidence of congenital rubella syndrome. Hereafter, eradication was achieved only once high coverage of gender-neutral MMR vaccination was achieved and followed by a strong herd effect. Measles, which has a high basic reproduction number (R0) of 10, was also essentially eradicated from the Nordic countries through this programme, and the status is still retained [4].

Among the more than 300 different HPVs infecting humans, types 16, 18, 31 and 45 are the most oncogenic viral agents for humans with over 90% of the HPV-associated cancer burden. The elimination of HPV-associated cancers will be accomplished by herd effect enforced eradication of these most oncogenic and prevalent HPV types. Because of both, the much lower R0, less than 4 for all other HPV types than HPV16, and the assortativeness of sexual risk-taking behaviour, it is the herd effect from moderate to high coverage gender-neutral vaccination that is the relevant factor providing protection even against HPV16 [5,6]. A community-randomised study has shown that the herd effect is notable in a real-life setting within less than 4 years but only after gender-neutral vaccination [5,6]. In addition, most mathematical models do not account for this, and thus their cost-efficiency estimates for the gender-neutral HPV vaccination could (should) be even better.

Colzani et al. [1] meticulously go through the European HPV vaccination policies. They contemplate whether or not it is morally right to prioritise offering HPV vaccine to girls in low- and middle-income countries (LMICs) with high cervical cancer incidence or if offering vaccination to the entire population of the young is morally right. Ortu et al. [2] report on low HPV vaccination coverage in another risk group, men who have sex with men. In this respect, it is of note that countries will always be left with particular group(s) of unvaccinated individuals. Herd effect, which stems rapidly and efficiently from gender-neutral HPV vaccination, also protects those risk groups with low uptake of the offered HPV vaccine [7]. The herd effect induced by even moderate gender-neutral vaccination coverage is thus crucial to protect the most vulnerable who are not vaccinated. The moral aspect here is that when advocating, accepting, or implementing any prophylactic vaccination policy, one is not only protecting the individual (ourselves) but the community as well (others).

Most importantly, it may not be possible to easily circumvent HPV vaccine shortage by changing the vaccination schedule or dosing. No HPV vaccine-induced protective antibody level has been defined and overall, the effect from one-dose vaccinations, aside from that with live vaccines, is unclear and assumes boostering which has its caveats (e.g. likely limited access to and availability of services) in the LMICs. Studies have shown that up to 10% of the 4-valent HPV vaccine recipients do not have neutralising or total HPV18 L1-VLP (virus-like particle) antibodies 7 to 12 years post vaccination [8,9], which explains the less than 100% vaccine efficacy against invasive cervical cancer [10]. Furthermore, high-quality health registry-based data showed that HPV vaccine efficacy against immediate cervical precancer (cervical intraepithelial neoplasm (CIN)3+ ) from one-dose vaccination is significantly worse than from three-dose vaccination [11]. Taken together, attempts to solve the moral dilemma caused by temporary vaccine shortage should not cause new moral conflicts.
